# Comparison of the Characteristics of Intubation Airway Barrier Devices Using a Simulated Airway Task Trainer

**DOI:** 10.7759/cureus.22817

**Published:** 2022-03-03

**Authors:** Nathan D Stuempfig, Sara Toulouie, Edward J Durant, Nur-Ain Nadir

**Affiliations:** 1 Emergency Medicine, Kaiser Permanente Central Valley, Modesto, USA; 2 Emergency Medicine, California Northstate University College of Medicine, Elk Grove, USA

**Keywords:** covid-19, simulation, protection, barrier, intubation

## Abstract

Background: With the advent of variant strains such as Delta and Omicron, there have been renewed concerns regarding transmission of the severe acute respiratory syndrome coronavirus 2 (SARS-CoV2) (coronavirus disease 2019 (COVID-19)) disease to healthcare professionals, particularly during intubation procedures. Several forms of barrier protection aimed at decreasing the spread of aerosolized droplets were developed during the early onset of the pandemic.

Objectives: Using a simulated airway model, we examined the impact that three separate barrier devices had on intubation time and success using both direct and video laryngoscopy. We hypothesized that the functionally simplistic devices would be preferred and would allow for faster intubations.

Methods: Just-in-time training sessions focusing on COVID-19 intubations were set up between March and June of 2020. Sixty-seven emergency physicians and anesthesiologists participated. For a subset of physicians, exact times to barrier device setup and both direct and indirect intubations using three different barrier devices were recorded. Subsequently, physicians were asked to fill out a survey regarding their experiences.

Results: The survey response rate was 60%. In general, this cohort preferred a plain clear plastic drape or clear plastic drape with polyvinyl chloride (PVC) cube for direct laryngoscopy and video laryngoscopy setups. The use of these two devices resulted in significantly faster times to completed intubation when compared with the fiberglass box while using a simulated task trainer.

Conclusion: In general, a simple, plastic sheet was the preferred barrier device using video laryngoscopy. Although setup times were faster using the fiberglass box, intubation times were significantly faster using the plastic drape or PVC frame.

## Introduction

With the advent of the severe acute respiratory syndrome coronavirus 2 (SARS-CoV2) pandemic, there have been significant concerns regarding the transmission of the disease to healthcare professionals (HCPs). This concern has increased as more virulent and transmissible strains such as Delta and Omicron have become the dominant cause of coronavirus disease 2019 (COVID-19) throughout the world. Due to the much higher degree of transmissibility, there is significant concern that the healthcare workforce may suffer from infection rates that could lead to serious staffing shortages, regardless of vaccination status [1-3]. Research suggests that SARS-CoV2 is mostly transmitted via 5-10 μ droplets, especially during aerosol-generating procedures (AGPs) and can become airborne in droplet nuclei less than 5 μ in size. These droplet nuclei can travel greater than 1 m, remain airborne for up to 3 hours, and have the potential to pass through the pores of surgical masks [4]. Some reports indicate that HCPs have acquired SARS-CoV2 infection despite the use of an N95 respirator [5]. Nevertheless, the Centers for Disease Control and Prevention (CDC) recommends that HCPs use a higher level of respiratory protection than N95 respirators alone, including eye protection, gowns, and gloves when performing AGPs [6]. AGPs such as supraglottic airway ventilation, intubation, extubation, and cardiopulmonary resuscitation generate aerosols of high viral loads leading to increased risk for HCPs [4,7,8].

Endotracheal intubation of SARS-CoV2 patients is a notably infectious procedure because it increases viral transmission among HCPs with an odds ratio of 8.8% when compared to unexposed providers [4,8,9,10]. With more than 8% of COVID-19 patients requiring intubation and up to 20% requiring multiple attempts, it is of paramount importance to enforce innovative protective barriers [8,11]. With the lack of FDA-approved intubation protection barriers and intermittent shortages of personal protective equipment (PPE) clinicians have been compelled to improvise protective barriers during AGPs [12]. Several barrier devices such as methyl methacrylate (plexiglass) hoods, nylon hoods, and clear plastic sheets have undergone preliminary testing and been unofficially recommended for limiting aerosol exposure [13,14]. Important criteria to consider when deciding on an appropriate barrier include the ease of airway access, containment of aerosolization, time required for setup, operator familiarity and comfort with device, and the intubation setting (e.g., emergent or controlled) [15]. Simulation training provides a safe environment for crisis management training and is currently being used to create novel protocols and devices to train HCPs for situations that disrupt traditional modes of care delivery [16]. In this study, we compare characteristics of three different airway barrier devices made from plastic drapes, polyvinyl chloride (PVC), or fiberglass through the use of a simulated airway task trainer and further compare the preference of practicing community emergency physicians and anesthesiologists for various barrier devices. In addition, we aim to determine which barrier devices allow for the fastest intubation times. This information may help to guide recommendations on usage of barrier devices during AGPs in COVID-19 patients and can be applied to training future HCPs who may need to perform intubations in emergent situations.

This article was previously presented as a meeting abstract at the 2021 CORD Academic Assembly on April 12, 2021, at the 2021 Society for Academic Emergency Medicine (SAEM) Annual Meeting on May 13, 2021, and at the International Meeting on Simulation in Healthcare (IMSH) Annual Meeting on January 11, 2022.

## Materials and methods

**Figure 1:**
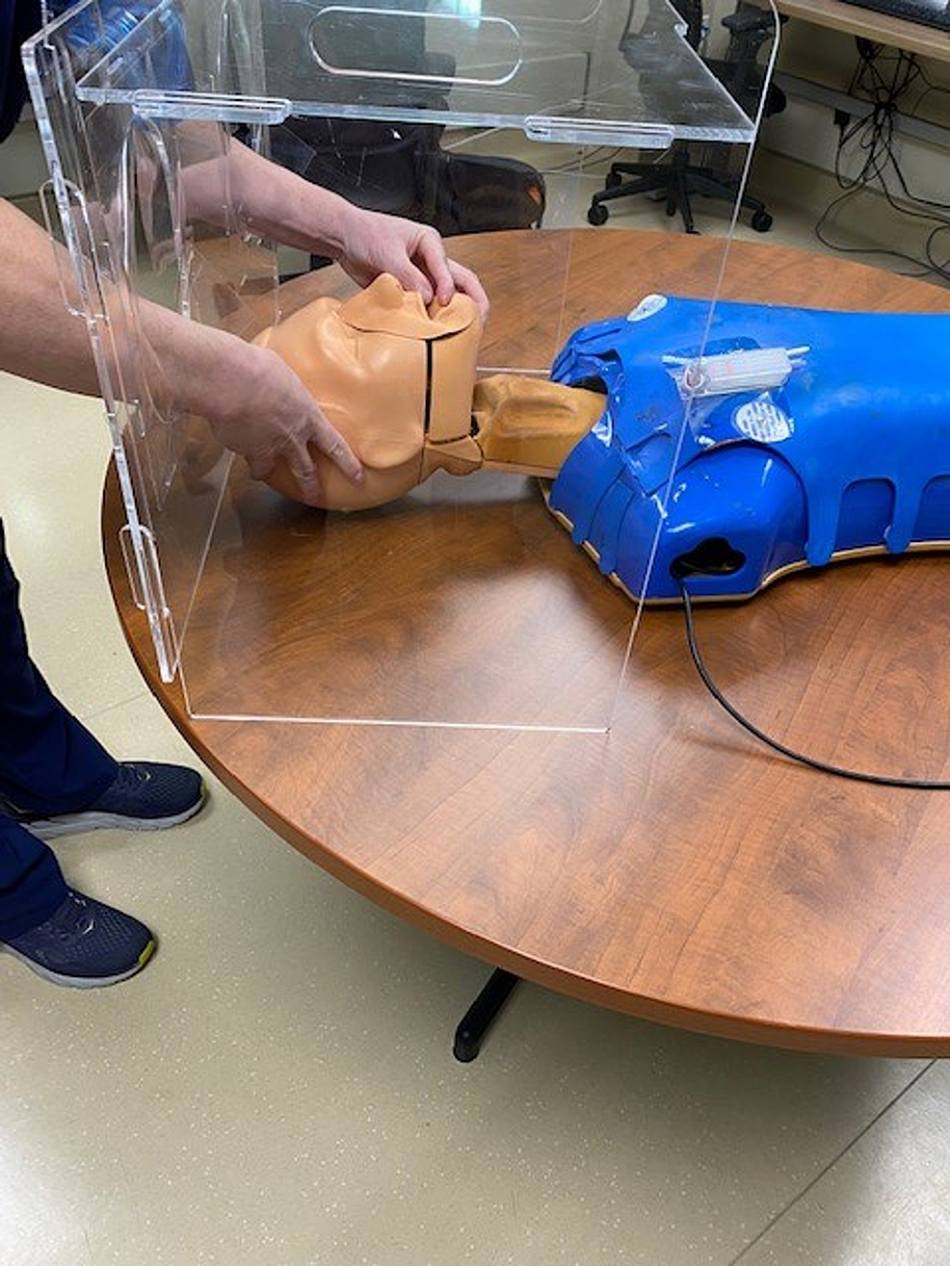
Clear plexiglass box

**Figure 2:**
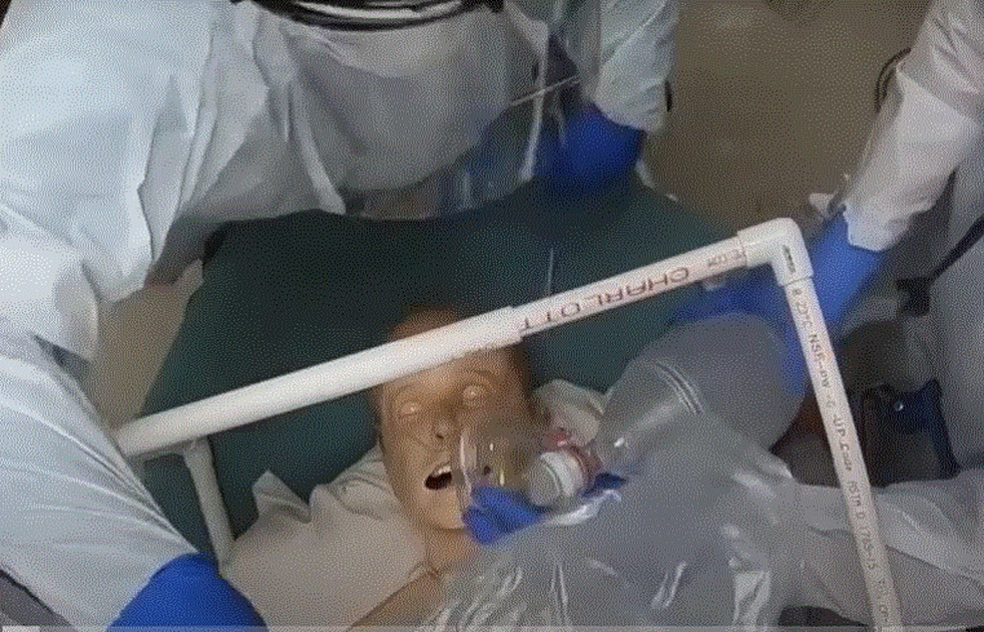
PVC frame with drape PVC: polyvinyl chloride

**Figure 3:**
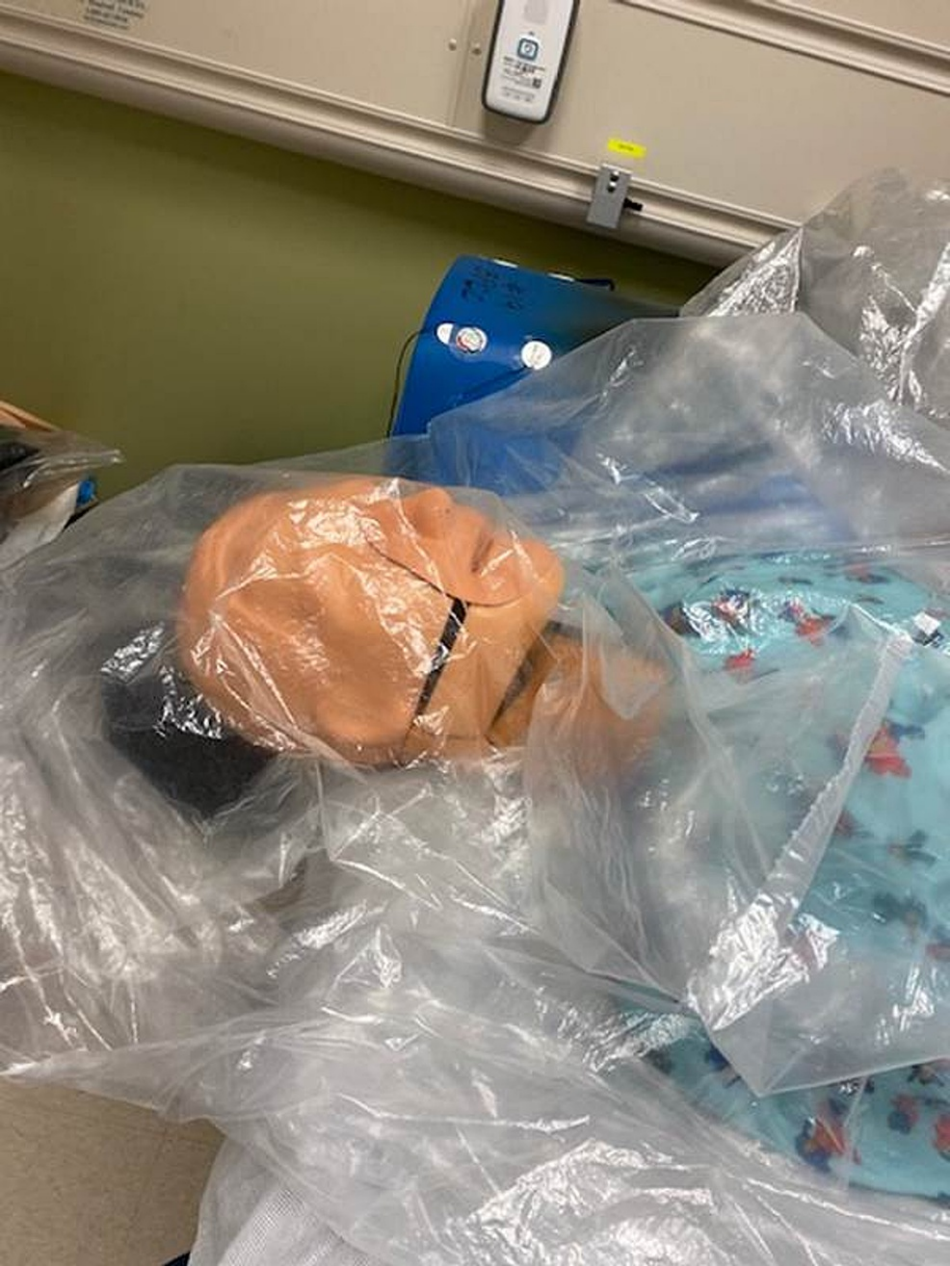
Plastic drape

This is a mixed-methods study examining the characteristics of three intubation barrier devices available to physicians in a 152-bed community hospital from April 2020 to June 2020. These devices consisted of a clear plexiglass box (Figure 1), a PVC-pipe cube with a plastic drape (Figure 2), and a clear plastic drape alone (Figure 3). Faculty in the department of anesthesia and emergency medicine were invited to participate in just-in-time training (JITT) sessions involving a mock code blue patient in which participants were offered the chance to experiment with all three devices while performing intubations on a manikin. JITT allows for practice of a skill immediately prior to performing a procedure in an effort to maximize trainee learning, confidence, and patient safety [17]. This study was deemed not research by our IRB.

Post-training survey

A survey instrument examining various aspects of COVID-19 specific intubation, including comfort with direct, indirect intubations, and comfort with barrier devices, was constructed and piloted among two emergency physicians. Feedback from the pilot was incorporated into the final version of the survey which was then circulated to all participants four weeks after the training. Narrative comments on the survey were further subjected to analysis and recurring themes identified.

Timed barrier device intubations

Emergency physicians were asked to intubate a simulated Laerdal^TM^ airway task trainer both by direct and video laryngoscopy while using each barrier device. Simple randomization was used to select the sequence of timed intubations for the three barrier device setups. The following measurements were calculated by two independent observers: time to device setup, time to first-pass intubation, and time to first bag valve mask (BVM). The procedure was considered complete when the BVM was attached to the successfully placed endotracheal tube. Obtained values were averaged. We calculated simple descriptive statistics, including mean time to set up, intubation, and ventilation for each device. We then performed a one-way analysis of variance (ANOVA) to compare the meantime to ventilation between devices, using both direct and indirect laryngoscopy.

## Results

**Figure 4:**
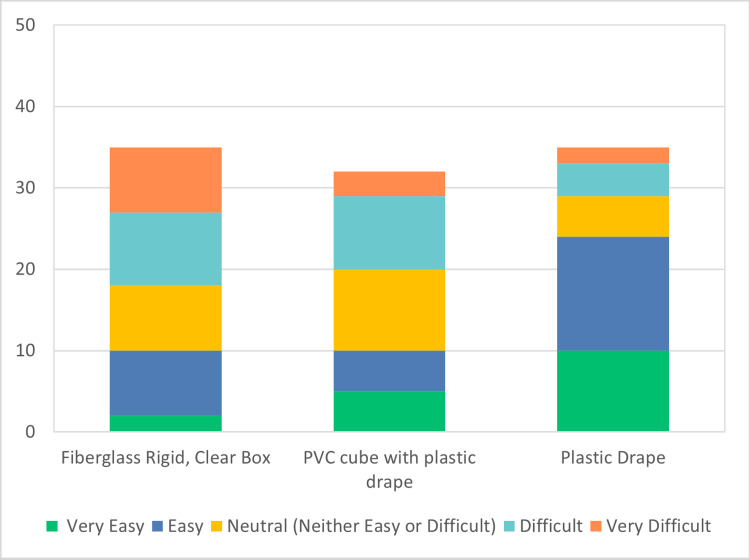
Ease of setup for direct laryngoscopy PVC: polyvinyl chloride

**Figure 5:**
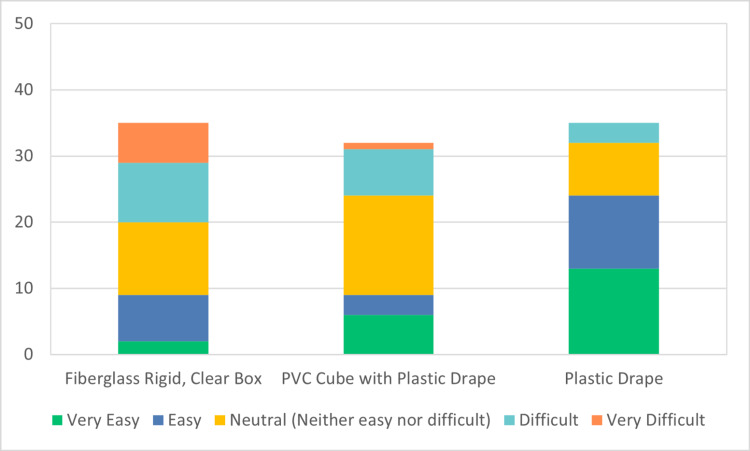
Ease of setup for video laryngoscopy PVC: polyvinyl chloride

**Figure 6:**
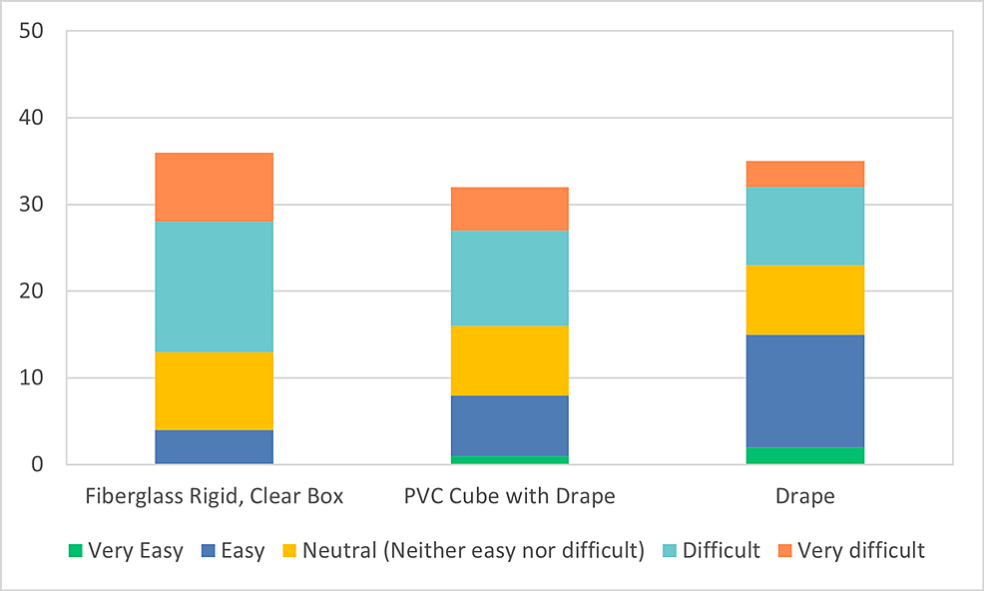
Ease of mastery using direct laryngoscopy PVC: polyvinyl chloride

**Figure 7:**
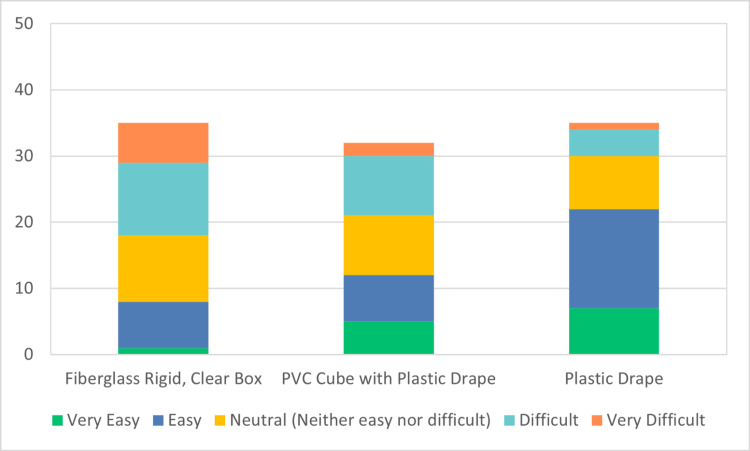
Ease of mastery using video laryngoscopy PVC: polyvinyl chloride

**Figure 8:**
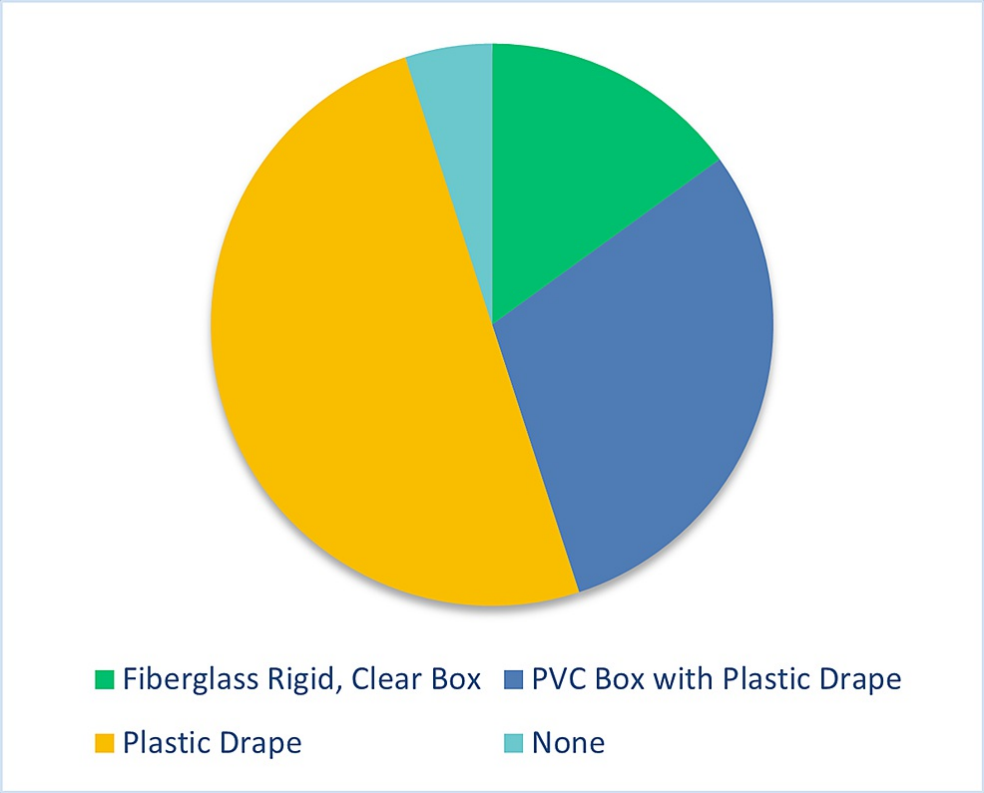
Preference of barrier device for any method of intubation PVC: polyvinyl chloride

A total of 67 participants participated in the JITT sessions. A total of 40 participants out of 67 responded to the survey, indicating a response rate of 60%. The results of the survey are depicted in Figures 4-7. 40% of the respondents were anesthesiologists and 60% emergency physicians. 50% of respondents had been out of residency training for more than 15 years. 60% of total respondents felt more comfortable with direct laryngoscopy; however, 97% of respondents indicated a preference for indirect or video laryngoscopy for COVID-19 positive or patient under investigation (PUI) patients. All respondents indicated familiarity and practice with one or more barrier devices. In general, this cohort preferred a plain clear plastic drape or clear plastic drape with PVC cube for direct laryngoscopy and video laryngoscope setups (Figure 8). Almost 40% of respondents indicated that the performance of video or direct laryngoscopy was perceived to be more difficult with the rigid fiberglass box.

A narrative analysis of learners’ comments indicated that the majority preferred the PVC cube with drape or the clear plastic drape for indirect laryngoscopy and the clear fiberglass box for direct laryngoscopy and that this had to do with visibility (or lack thereof) through the clear drape which is more essential for direct laryngoscopy. A preference for direct laryngoscopy and the clear fiberglass box also correlates with participants who did not traditionally train with video laryngoscopy, such as those who graduated from residency more than 10 years ago. When barrier devices were available, a majority of respondents preferred the clear plastic drape or the PVC box with the overlying drape.

**Figure 9:**
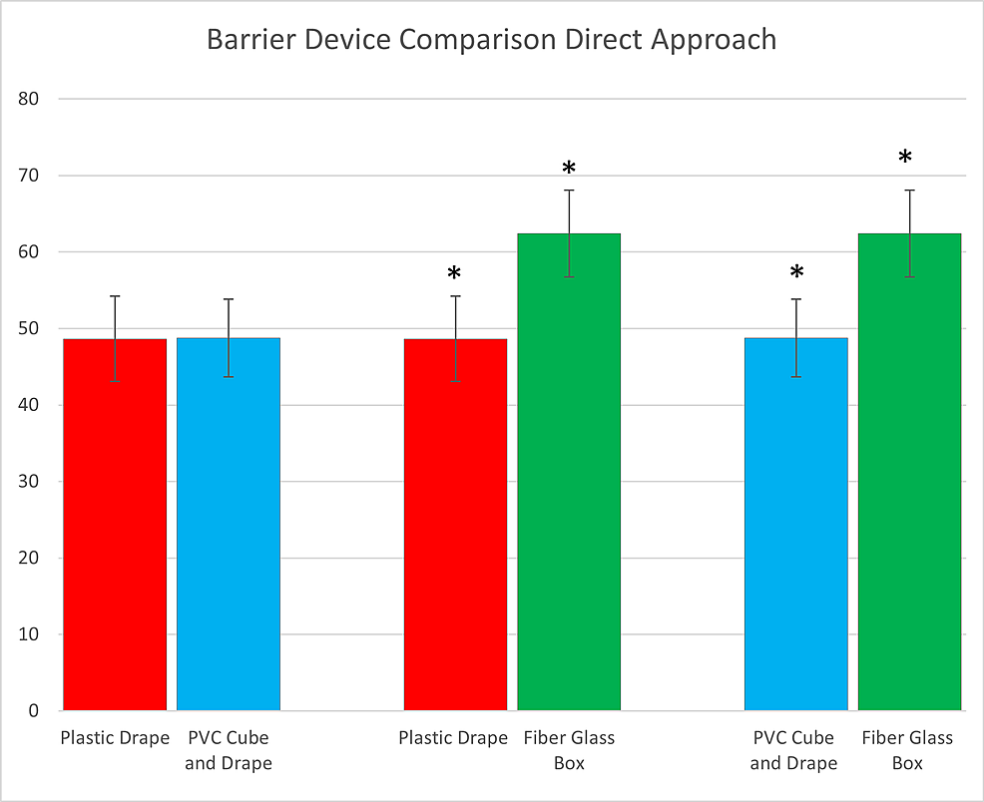
Direct comparison of barrier devices for average completed intubation and ventilation times using direct laryngoscopy *demonstrates statistical significance PVC: polyvinyl chloride

**Figure 10:**
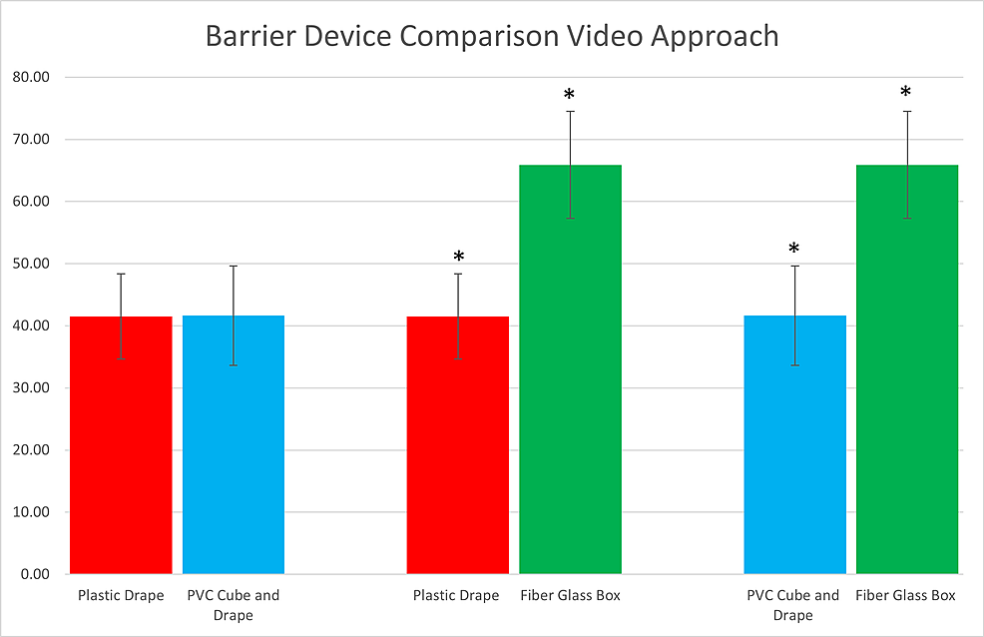
Direct comparison of barrier devices for average complete intubation and ventilation times using video laryngoscopy *demonstrates statistical significance PVC: polyvinyl chloride

With respect to the barrier device setups, 10 physicians participated in the specific timed trial measurements. Results depicted in Tables 1-2, as well as Figures 9-10, show that there were statistically significant differences between barrier devices. Set up of the fiberglass box was faster compared to the setup of the plastic drape or the PVC cube plus overlying plastic drape for both direct and video laryngoscopy. However, the average time from the start of the procedure to successful intubation and ventilation was faster using either the plastic drape or PVC cube plus drape when compared to the fiberglass box using both indirect and video laryngoscopy. There were no statistically significant time differences between the plastic drape or the PVC cube with an overlying drape.

**Table 1:** Average times to barrier device set up, 1st pass intubation, and BVM with direct and indirect laryngoscopy for each intubation barrier device. BVM: bag valve mask; PVC: polyvinyl chloride

Barrier device type	Plastic drape		PVC cube with plastic drape		Fiberglass box	
	Direct	Indirect	Direct	Indirect	Direct	Indirect
Average time (s)						
Device setup	32.4 + 4.12	34 + 4.12	40 + 8.1	38.7 + 6.1	11.3 + 1.59	9.5 + 2.07
1^st^ pass intubation	46 + 4.4	38.6 + 3.96	45.5+ 4.5	41 + 4.3	56.5 + 7.4	52 + 3.9
BVM	48.6 + 5.57	41.5 + 6.8	48.8 + 5.1	42.8 + 5.9	63 + 5.6	65 + 8.6

**Table 2:** Direct comparison of barrier devices for average complete intubation and ventilation *denotes statistical significance PVC: polyvinyl chloride

Laryngoscopy approach	Barrier device comparison	Setup to intubation/ventilation times (P-value)
Direct	Plastic drape vs PVC cube and drape	0.963
Direct	Plastic drape vs fiberglass box	<0.001*
Direct	PVC cube and drape vs fiberglass box	<0.001*
Video	Plastic drape vs PVC cube and drape	0.974
Video	Plastic drape vs fiberglass box	<0.001*
Video	PVC cube and drape vs fiberglass box	<0.001*

## Discussion

Our study examined the characteristics of three barrier devices with respect to perceived ease of usage, time of intubation or device set up, and time to complete intubation and ventilation. In addition, we were able to evaluate the impact any of these barrier devices had on the ability to quickly and successfully perform intubations which has the potential to affect patient outcomes [18]. Although there have been several studies examining the logistics and feasibility of various barrier devices, our study additionally explores the usability of these devices from the perspective of physicians who are most likely to use them. The use of a protective drape or patient cover does not replace the need for, nor does it decrease the importance of appropriate PPE for all healthcare workers during potentially aerosolizing procedures [19]. However, it can provide additional protection to healthcare workers during AGPs such as intubation. As can be expected, there is a steep learning curve to the incorporation of any barrier device when intubating.

Based on our results, the plexiglass box appears to be the least user-friendly for purposes of both direct and indirect laryngoscopy, even though it allows for a short setup time. Narrative comments provided during JITT sessions indicated that lack of space to maneuver equipment and decreased arm range of motion were the most common negative perceptions. When compared to the other devices tested, clinicians not only felt less comfortable using the plexiglass box but were slower to perform intubations with the use of this device when compared to the other tested devices. Although these devices were tested in a simulated environment, we suspect that these negative perceptions would be exacerbated in a real code situation where the environment is far less controlled. Further, this study demonstrates that with minimal training, a majority of clinicians prefer to use straightforward clear plastic drapes when intubating, particularly while performing video laryngoscopy. Narrative comments during JITT sessions indicated that clear plastic drapes allowed for a greater range of motion and easy maneuverability. In addition, a simple clear plastic drape or plastic drape with PVC frame allowed for faster total intubation times of the airway task trainer when compared to the prefabricated plexiglass box.

Simple plastic drapes are readily available in most emergency departments, hospital floors, and operating rooms. They do not require any construction materials and are relatively inexpensive when compared to other prefabricated intubation barrier devices. Given the easy accessibility and decreased costs of simple plastic drapes, it may be worthwhile to consider them more extensively for intubation purposes in situations where aerosolized particles remain a concern. With the development of extremely transmissible COVID-19 variants and the potential rise of other highly contagious viral diseases, healthcare worker protection is of paramount importance, particularly while performing intubations. Plastic drapes are easily accessible, inexpensive, and user- friendly. Their use during intubation should be strongly considered in order to help ensure HCP safety.

Limitations

The use of manikins in this study may pose inevitable limitations to clinical application as there are important discrepancies in the upper airway anatomy along with variations in the impact peak pressure, tidal volume, and leakage measurements. Additionally, researchers should evaluate the characteristics of their manikin to consider its intrinsic resistance and airway dead space for the definition of their patient model. Other limitations to our study include a small sample size (n=67, n=10) limited to Stanislaus County in a single hospital setting. The time-compressed nature of JITT hindered the ability to increase the sample size of the study and prevented us from collecting data across multiple medical centers. In addition, an individual clinician’s previous experience with a particular barrier device may alter intubation times and preference of use.

## Conclusions

The application of a barrier device has been shown to reduce the transmission of aerosolized viral particles. Variant strains of the COVID-19, such as Omicron, have shown to have much higher degrees of transmissibility when compared to the original COVID-19 virus. This has led to an increase in the need for mitigating the potential transmission of COVID-19 to HCPs. These barrier devices can be inexpensive and their use can be quickly adapted. Our study shows that a simple, plastic drape, which is readily available in nearly every emergency department, can be used to increase the safety of HCPs without impacting procedural performance. In this study, physicians not only preferred to use a more simplistic barrier but also performed intubations more quickly when compared to purchased, prefabricated devices.
